# Postnatal Development of Prospective Beef Bulls: Integrating Nutrition, Metabolism, Endocrine Regulation, and Breeding Soundness

**DOI:** 10.3390/biology15171522

**Published:** 2026-09-03

**Authors:** Borhan Shokrollahi, Jae-Yong Choi, Sang-Min Shin, Seung-Eun Lee, Miyoung Won

**Affiliations:** Subtropical Livestock Research Center, National Institute of Animal Science, Rural Development Administration (RDA), Jeju 63242, Republic of Korea; borhansh@gmail.com (B.S.); jaechoi@korea.kr (J.-Y.C.); adamrib@korea.kr (S.-M.S.); selee81@korea.kr (S.-E.L.)

**Keywords:** postnatal development, beef sire, breeding soundness, nutrient sensing, metabolic adaptation, endocrine regulation, precision phenotyping, systems biology, reproductive development, precision livestock farming

## Abstract

Postnatal development in developing beef bulls is regulated through coordinated interactions among nutritional, metabolic, endocrine, and physiological processes that collectively determine structural maturation, reproductive competence, and breeding soundness. Optimizing these integrated regulatory mechanisms is essential for improving reproductive efficiency, accelerating genetic gain, and enhancing the sustainability of beef production systems. This review synthesizes current knowledge on the integrated regulation of postnatal development in developing beef bulls by combining evidence from developmental physiology, nutrition, metabolism, endocrine regulation, and reproductive biology. Particular emphasis is placed on tissue-specific maturation, nutrient-sensing and endocrine signaling, metabolic adaptation, developmental nutrient partitioning, pubertal development, and the biological mechanisms underlying breeding soundness. The review further evaluates practical approaches for assessing developmental progress using growth and structural traits, metabolic and endocrine biomarkers, reproductive indicators, and breeding soundness evaluation while highlighting the emerging roles of precision nutrition, precision phenotyping, multi-omics, wearable sensor technologies, artificial intelligence, and predictive modelling in developmental management. Current evidence indicates that postnatal development is governed by integrated biological networks rather than isolated physiological processes. Adoption of systems biology and data-driven approaches will facilitate earlier prediction of developmental competence, enable precision management of replacement sires, and improve reproductive efficiency, genetic progress, and the long-term sustainability of beef production.

## Simple Summary

The development of future beef breeding bulls begins long before they produce semen or enter breeding programs. Nutrition, growth, metabolism, hormones, and body development work together from birth to determine whether a young bull will become a healthy and fertile breeding animal. Understanding how these biological processes interact is important for improving reproductive performance, animal health, and the long-term sustainability of beef production. This review explains how proper nutrition supports normal growth, how metabolism converts nutrients into energy and body tissues, and how hormones coordinate physical and reproductive development during the different stages of postnatal life. It also discusses new approaches that combine biological measurements, advanced monitoring technologies, and artificial intelligence to evaluate developmental progress and identify superior breeding bulls earlier than traditional methods. By bringing together current knowledge from different areas of animal biology, this review provides an integrated understanding of how young beef bulls develop from birth to breeding age. This information can help researchers, veterinarians, nutritionists, and cattle producers improve management practices, increase reproductive efficiency, and support more sustainable beef production systems.

## 1. Introduction

Beef cattle production is undergoing substantial transformation in response to increasing demands for sustainable livestock systems, improved production efficiency, and accelerated genetic gain. Advances in genomic selection, reproductive biotechnologies, precision nutrition, and precision livestock management are creating new opportunities to enhance the biological and economic efficiency of beef production. Within this context, reproductive efficiency remains a major determinant of herd productivity, profitability, and sustainability because the number and performance of calves directly influence production efficiency and economic returns [1,2]. Although considerable emphasis has traditionally been placed on female fertility, breeding bulls exert a disproportionately large influence on herd-level reproductive performance and genetic progress because a single sire can produce offspring from numerous females [3,4,5]. Consequently, developing genetically superior breeding bulls has become a cornerstone of modern beef breeding programs, making optimization of postnatal development, reproductive competence, and lifetime fertility a strategic priority for sustainable beef production systems [3,6].

Unlike beef steers, which are managed primarily to maximize growth performance, feed efficiency, carcass yield, and meat quality, developing bulls follow a fundamentally different developmental trajectory in which skeletal development, sexual maturation, reproductive capacity, structural soundness, and long-term breeding performance are the primary management objectives [3,7,8]. Successful sire development, therefore, depends on the integrated regulation of postnatal development, resulting in the coordinated establishment of structural, metabolic, endocrine, immune, and reproductive competence rather than rapid body weight gain alone [3,7,9,10]. Rather than functioning as independent physiological processes, nutrition, metabolism, endocrine regulation, tissue maturation, and reproductive development form an interconnected biological network that collectively determines developmental competence and ultimately breeding soundness. Perturbations during critical developmental windows can permanently alter developmental trajectories, affecting pubertal development, semen production, fertility, longevity, and the efficient dissemination of superior genetics [7,11].

Postnatal development is driven by the continuous partitioning of nutrients and metabolic resources among competing physiological processes, including skeletal growth, muscle accretion, visceral organ maturation, immune function, adipose tissue deposition, and reproductive development [12,13,14,15]. The efficiency of this resource allocation influences not only somatic growth but also metabolic adaptation, endocrine maturation, pubertal timing, testicular development, and future reproductive capacity [7,10]. Early-life nutrition is increasingly recognized as a critical regulator of developmental programming because it modulates nutrient-sensing, metabolic, and neuroendocrine signaling pathways that exert persistent effects on tissue maturation, reproductive development, and lifetime fertility [16,17]. These effects are mediated through integrated regulatory networks, including the growth hormone–insulin-like growth factor-1 (GH–IGF-1) axis, insulin, leptin, nutrient-sensing pathways, and the hypothalamic–pituitary–gonadal (HPG) axis, which coordinate somatic growth with reproductive maturation [16,17,18]. Recent advances in genomics, transcriptomics, epigenetics, metabolomics, and other multi-omics technologies have further demonstrated that molecular regulation contributes to the developmental plasticity of young bulls, creating new opportunities for precision nutrition, genomic selection, and reproductive management [17,19]. Collectively, these observations indicate that postnatal development should be viewed as an integrated regulatory process rather than a series of independent developmental events.

Despite substantial advances in nutrition, reproductive physiology, breeding soundness evaluation, developmental biology, and molecular sciences, current knowledge remains fragmented, with these disciplines often investigated independently rather than as components of an integrated developmental system [3,5,7,9,20]. Consequently, a comprehensive conceptual framework describing the integrated regulation of postnatal development from birth to breeding soundness remains lacking. Existing knowledge is largely organized according to individual disciplines, including nutrition, metabolism, endocrinology, reproductive physiology, and molecular biology, rather than as interacting biological systems that collectively determine developmental competence [3,5,7,9]. This fragmentation limits our understanding of how interconnected regulatory mechanisms shape postnatal development and hinders the translation of emerging biological knowledge into practical breeding and management strategies.

At the same time, precision livestock farming, genomic selection, high-throughput phenotyping, multi-omics technologies, and artificial intelligence are transforming the evaluation and selection of replacement breeding bulls [21,22,23]. These technologies enable continuous characterization of developmental trajectories, earlier prediction of reproductive potential, identification of biomarkers associated with breeding soundness, and implementation of precision nutritional and management strategies [6,17,21]. Integrating these emerging technologies with developmental physiology and molecular biology will be essential for designing next-generation breeding strategies that improve productivity, reproductive efficiency, and sustainability.

This review presents an integrated framework describing the regulation of postnatal development in developing beef bulls from birth to breeding soundness. We examine how nutritional, metabolic, endocrine, molecular, and physiological regulation interact throughout postnatal life to coordinate tissue maturation and establish structural, metabolic, endocrine, and reproductive competence. Particular emphasis is placed on tissue-specific maturation, nutrient sensing, metabolic adaptation, endocrine regulation, pubertal development, breeding soundness, and emerging precision technologies that support earlier prediction and management of genetically superior breeding sires.

## 2. Biological Foundations of Postnatal Development in Developing Beef Bulls

### 2.1. Postnatal Development as a Coordinated Biological Process

**Figure 1:**
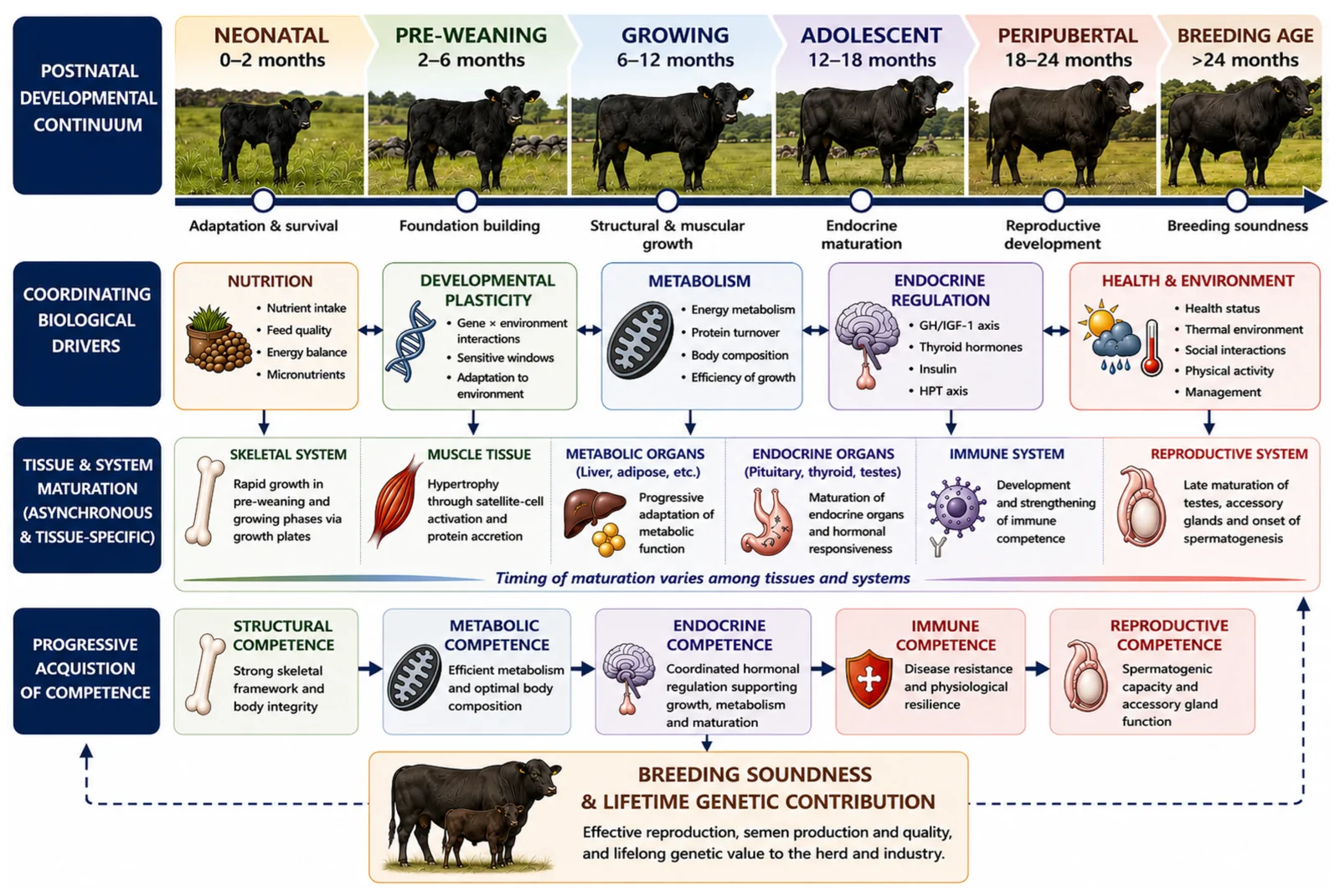
Conceptual framework illustrating the integrated regulation of postnatal development in developing beef bulls from birth to breeding soundness. Nutrition, developmental plasticity, metabolism, endocrine regulation, tissue maturation, and reproductive development interact throughout postnatal life to establish structural, metabolic, endocrine, immune, and reproductive competence, ultimately determining breeding soundness and lifetime genetic contribution.

Postnatal development of developing beef bulls is increasingly recognized as a coordinated biological process rather than simply the accumulation of body mass [7,10]. It is regulated through dynamic interactions among genetic potential, developmental plasticity, nutrition, metabolism, endocrine signaling, tissue-specific maturation, and environmental influences that collectively establish structural, metabolic, endocrine, immune, and reproductive competence [8,11,14]. Consequently, successful postnatal development depends not only on body weight or growth rate but also on the progressive acquisition of biological functions that ultimately determine breeding soundness, reproductive efficiency, and lifetime genetic value (Figure 1).

Although closely related, growth and development are distinct biological processes. Growth refers to quantitative increases in body mass, whereas development encompasses tissue differentiation, structural organization, and functional specialization leading to organ maturation [24,25]. In developing beef bulls, postnatal development extends beyond somatic growth to include the coordinated establishment of structural integrity, metabolic homeostasis, endocrine function, immune competence, and reproductive capacity, with these systems maturing concurrently but asynchronously according to changing physiological priorities [7,8,11].

A defining feature of postnatal development is tissue-specific maturation. Early postnatal life prioritizes neonatal adaptation, immune competence, gastrointestinal maturation, and maintenance of vital organ function [26,27]. During the pre-weaning and growing phases, skeletal growth establishes the structural framework for subsequent muscle accretion, while skeletal muscle develops primarily through satellite cell-supported hypertrophy [15,25]. Reproductive tissues mature later, with activation of the HPG axis initiating rapid testicular growth, accessory sex gland maturation, and spermatogenesis during the peripubertal period [3,10]. This asynchronous sequence reflects biological prioritization rather than uniform tissue growth and ensures that physiological resources are allocated according to stage-specific developmental requirements.

These developmental transitions are coordinated through hierarchical nutrient partitioning, whereby physiological resources are distributed among competing biological processes according to developmental stage. Maintenance and immune function dominate early life, followed sequentially by skeletal growth, muscle accretion, metabolic adaptation, endocrine maturation, and reproductive development as puberty approaches [14,26]. Developmental trajectories remain highly plastic, being shaped by interactions among genetic potential, nutrition, health status, thermal environment, and management practices [8,14,27,28,29]. Conversely, nutritional deficiency, disease, or chronic stress during critical developmental windows may permanently impair skeletal and testicular development, delay puberty, reduce semen production, and compromise lifetime reproductive performance.

At the cellular level, postnatal development progresses from rapid proliferation toward tissue-specific differentiation, hypertrophy, extracellular matrix remodeling, and functional maturation [15,28,30]. Skeletal muscle growth depends primarily on hypertrophy of existing fibers supported by satellite cells, whereas longitudinal bone growth occurs through chondrocyte proliferation, hypertrophy, and endochondral ossification within the epiphyseal growth plate [15,30,31]. Likewise, reproductive maturation requires coordinated differentiation of Sertoli, Leydig, and germ cells together with maturation of the seminiferous tubules and accessory sex glands, ultimately establishing reproductive competence and breeding soundness [11,32]. Collectively, these interconnected developmental processes provide the biological foundation upon which nutritional, metabolic, endocrine, and reproductive regulation act throughout postnatal life, supporting the progressive establishment of developmental competence from birth to breeding soundness.

### 2.2. Developmental Stages and Physiological Priorities from Birth to Breeding Age

**Figure 2:**
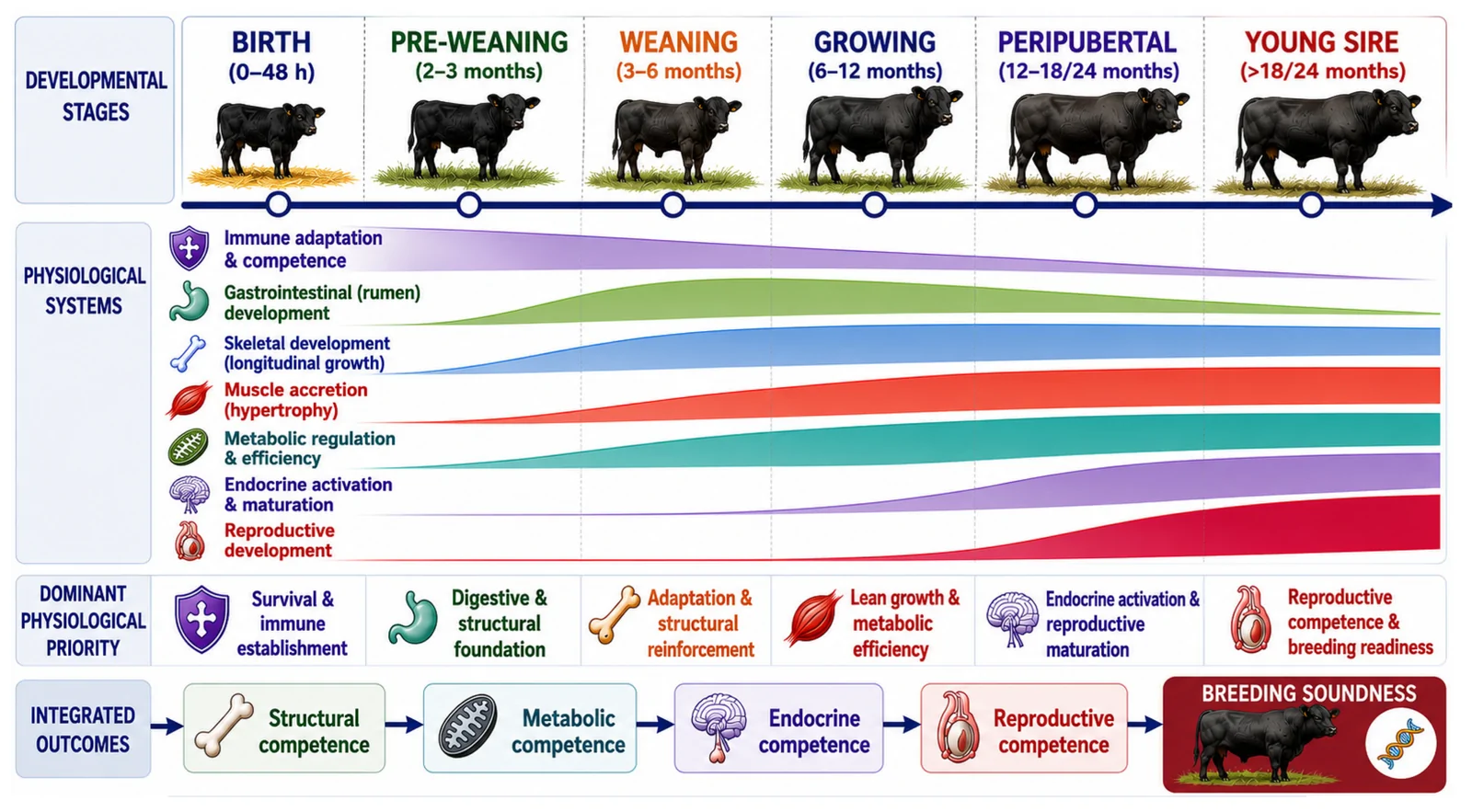
Developmental trajectory and shifting physiological priorities during postnatal development of developing beef bulls. The relative importance of immune adaptation, gastrointestinal maturation, skeletal growth, muscle accretion, metabolic regulation, endocrine activation, and reproductive development changes continuously from birth to breeding age, driving the sequential establishment of structural, metabolic, endocrine, and reproductive competence that ultimately leads to breeding soundness.

Postnatal development in developing beef bulls is a continuous and integrated process characterized by stage-specific physiological priorities and tissue-specific maturation rather than discrete developmental phases [11,28]. Dynamic nutrient partitioning, metabolic adaptation, and endocrine regulation sequentially establish structural, metabolic, endocrine, immune, and reproductive competence according to the changing biological requirements of the developing animal [26,33,34]. Understanding these developmental priorities provides the biological basis for nutritional and management strategies that optimize postnatal development while preserving structural integrity, reproductive competence, and long-term breeding soundness [3,8]. Representative physiological milestones are summarized in Table 1, and the developmental continuum is illustrated in Figure 2.

The neonatal period represents the most physiologically vulnerable stage, during which calves transition from intrauterine to extrauterine life [35]. Immediate priorities include thermoregulation, cardiopulmonary adaptation, passive immune transfer through colostrum, and gastrointestinal maturation [33,36]. Colostrum provides immunoglobulins that are essential for passive immune transfer and neonatal immune protection. In addition, colostrum contains hormones, cytokines, growth factors, and other bioactive compounds that contribute to gastrointestinal maturation, metabolic adaptation, immune maturation, and early growth [37,38]. During this stage, nutrients are preferentially allocated to maintenance, thermoregulation, immune function, and vital organ development rather than rapid tissue accretion [33,38].

Following neonatal adaptation, developmental priorities shift toward rumen maturation, skeletal growth, and lean tissue accretion, establishing the structural and metabolic foundation for subsequent endocrine activation and reproductive development [30]. As calves transition from milk to solid feed, increased substrate availability promotes ruminal microbial fermentation and the production of volatile fatty acids (VFAs), particularly acetate, propionate, and butyrate, which contribute to rumen epithelial maturation and the transition toward ruminant metabolism. Butyrate promotes rumen papillae development, whereas propionate increasingly supports hepatic gluconeogenesis and metabolic adaptation [15,39]. Concurrently, longitudinal bone growth and skeletal muscle hypertrophy accelerate through growth plate activity, satellite cell activation, and protein accretion [15,39]. Successful weaning therefore depends on adequate rumen development, appropriate nutrition, and effective management of weaning-associated stress, particularly the simultaneous effects of maternal separation and dietary transition. These stressors may reduce feed intake and growth and disrupt metabolic and immune adaptation during the transition to independent feeding [39,40].

The growing phase is characterized by rapid lean tissue accretion and continued musculoskeletal development, accompanied by progressive metabolic and endocrine maturation in preparation for puberty [7,41]. Balanced nutrition is essential because excessive energy intake promotes adipose deposition without proportionally improving skeletal or reproductive development, potentially compromising future breeding soundness [10,42].

As puberty approaches, developmental priorities progressively shift from somatic growth toward reproductive maturation. Activation of the HPG axis stimulates rapid testicular growth, differentiation of Sertoli and Leydig cells, initiation of spermatogenesis, and increasing testosterone secretion [7,9]. Although skeletal and muscular development continue, endocrine regulation increasingly coordinates reproductive maturation. Pubertal timing is influenced by genotype, nutrition, growth trajectory, metabolic and health status, and environmental conditions, highlighting the integrated regulation of postnatal development [43].

During the postpubertal period, continued maturation improves semen quality, sperm production, endocrine function, sexual behavior, and musculoskeletal development required for successful natural service [3,11,44]. At this stage, breeding capacity reflects the coordinated establishment of structural, metabolic, endocrine, behavioral, and reproductive competence rather than growth performance alone [11,45]. Accordingly, management should emphasize maintaining optimal body condition, preventing excessive adiposity, and preserving reproductive health to maximize breeding longevity and lifetime genetic contribution.

### 2.3. Tissue-Specific Development and Functional Maturation

**Figure 3:**
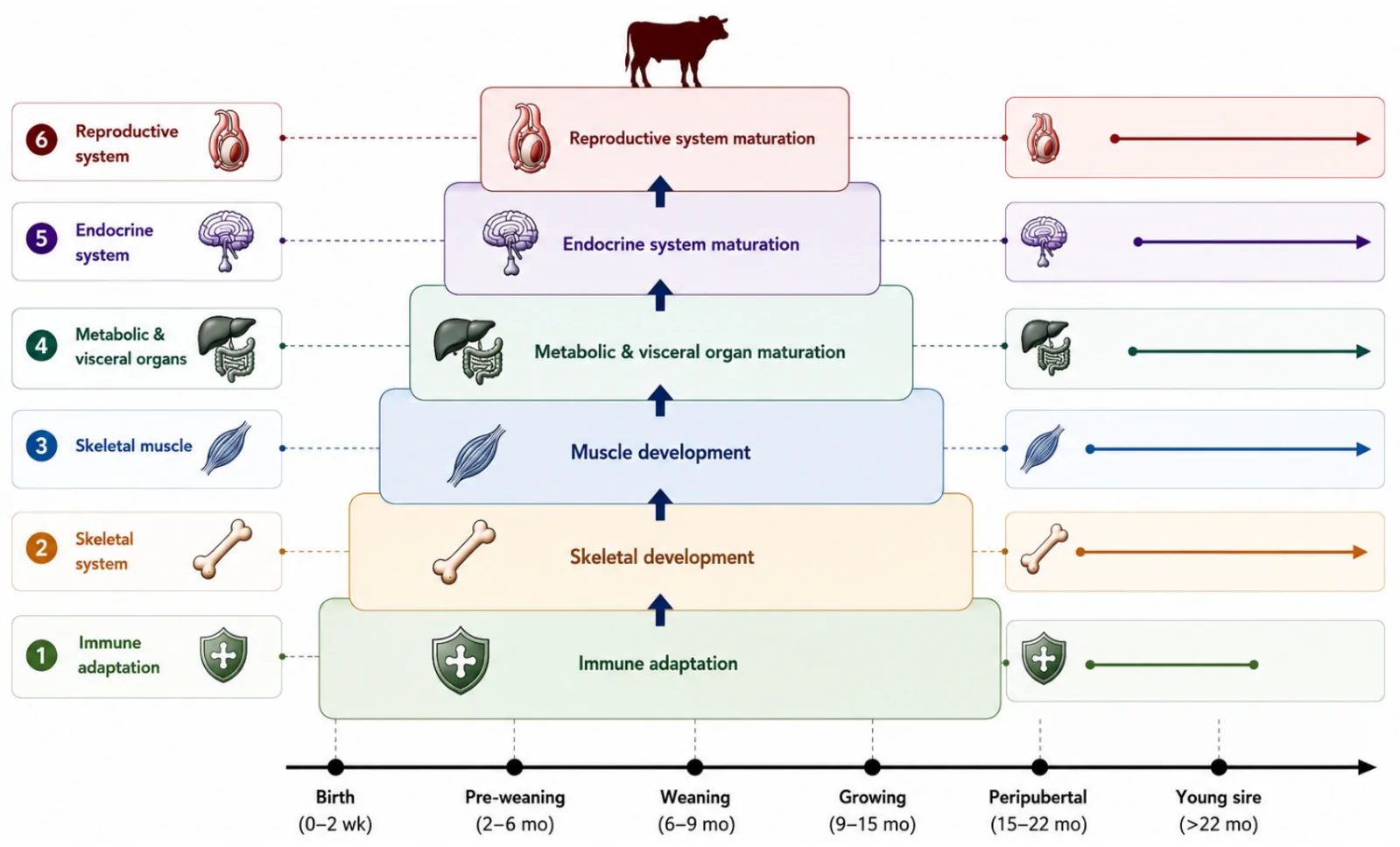
Tissue-specific maturation during postnatal development of developing beef bulls. Major physiological systems mature asynchronously from birth to breeding age. Immune adaptation predominates during early neonatal life, followed by sequential skeletal, muscular, metabolic, endocrine, and reproductive maturation. Coordinated maturation of these interconnected systems progressively establishes structural, metabolic, endocrine, and reproductive competence, ultimately leading to breeding soundness.

A defining feature of postnatal development in developing beef bulls is the asynchronous maturation of tissues and organ systems. Skeletal, muscular, gastrointestinal, endocrine, and reproductive tissues follow distinct but coordinated developmental trajectories that reflect changing physiological priorities and dynamic nutrient partitioning, leading to the progressive establishment of structural, metabolic, endocrine, and reproductive competence [26,28,46,47] (Figure 3). Therefore, body weight or chronological age alone provides an incomplete assessment of developmental progress because tissues differ markedly in their rates of growth, maturation, and functional specialization.

#### 2.3.1. Skeletal Development and Structural Competence

Skeletal development is prioritized during early postnatal life because the skeleton provides the structural framework for muscle accretion, locomotion, and reproductive performance [44,48]. Longitudinal bone growth occurs primarily through endochondral ossification at the epiphyseal growth plates, accompanied by continuous remodeling and mineralization [49,50]. Rapid skeletal development during the growing phase establishes the structural support required for increasing body mass and efficient locomotion [44]. Adequate skeletal and musculoskeletal development contributes to structural soundness, locomotor function, and the physical competence required of breeding bulls, thereby representing an important component of overall breeding soundness [48,51,52].

#### 2.3.2. Skeletal Muscle Development and Functional Capacity

Skeletal muscle becomes the principal site of postnatal tissue accretion and accounts for most lean tissue growth [31,53]. Because muscle fiber number is largely established before birth, postnatal muscle development depends predominantly on hypertrophy of existing fibers supported by satellite-cell activation, myonuclear accretion, and protein synthesis [12,54]. Beyond contributing to body growth, skeletal muscle plays a central role in locomotion, glucose and amino acid metabolism, and whole-body energy homeostasis, providing the functional capacity and metabolic resilience required for sustained breeding activity [31,41,54]. Consequently, balanced muscle development supports both structural performance and metabolic competence throughout postnatal development.

#### 2.3.3. Adipose Tissue Development and Body Composition

Adipose tissue matures later than skeletal and muscular tissues, with substantial fat deposition occurring after the principal phases of structural growth [55,56]. Moderate adiposity contributes to energy storage, endocrine regulation, and metabolic homeostasis, whereas excessive energy intake and adiposity may adversely affect metabolic regulation and reproductive development. Recent studies in young beef bulls have associated overnutrition and increased adiposity with alterations in semen characteristics, although these relationships do not necessarily indicate a direct causal effect of scrotal fat accumulation on fertility [57,58,59,60]. Therefore, body composition provides a more informative indicator of developmental progress than body weight alone by distinguishing appropriate lean tissue accretion from excessive adiposity.

#### 2.3.4. Gastrointestinal and Visceral Organ Maturation

Functional maturation of the gastrointestinal tract and associated visceral organs underpins progressive improvements in nutrient digestion, absorption, and metabolic utilization [30,61]. The rumen undergoes one of the most profound developmental transitions as calves shift from preruminant digestion to microbial fermentation following solid feed intake [61,62]. Concurrent maturation of the liver, pancreas, kidneys, and other visceral organs enhances nutrient metabolism, endocrine regulation, metabolic flexibility, and whole-body homeostasis [28,30,63,64]. Together, these coordinated adaptations establish the physiological foundation for efficient nutrient utilization, metabolic competence, and subsequent reproductive development [7,30].

#### 2.3.5. Development of the Male Reproductive System

The male reproductive system is among the last organ systems to reach functional maturity. During early postnatal life, testicular growth remains limited while physiological resources are preferentially allocated to structural growth and metabolic development [11,46]. As puberty approaches, activation of the HPG axis drives rapid maturation of the testes, epididymis, and accessory sex glands, increasing testosterone secretion and establishing the anatomical and endocrine basis for spermatogenesis [7,11,46]. Progressive differentiation of Sertoli, Leydig, and germ cells creates the cellular environment required for sperm production and reproductive competence [65]. Although reproductive tissues mature later than skeletal and muscular systems, their functional development depends on the prior establishment of structural integrity, metabolic competence, and endocrine regulation, emphasizing the integrated regulation of postnatal development [11,28,46]. Consequently, breeding soundness represents the culmination of coordinated maturation across multiple physiological systems rather than reproductive development alone [11,28,33,66]. The major characteristics of tissue-specific maturation and their contributions to breeding soundness are summarized in Table 2, providing the biological foundation for the subsequent sections on nutritional, metabolic, and endocrine regulation of postnatal development [7,11,66].

## 3. Nutritional Regulation of Growth and Development in Developing Beef Bulls

**Figure 4:**
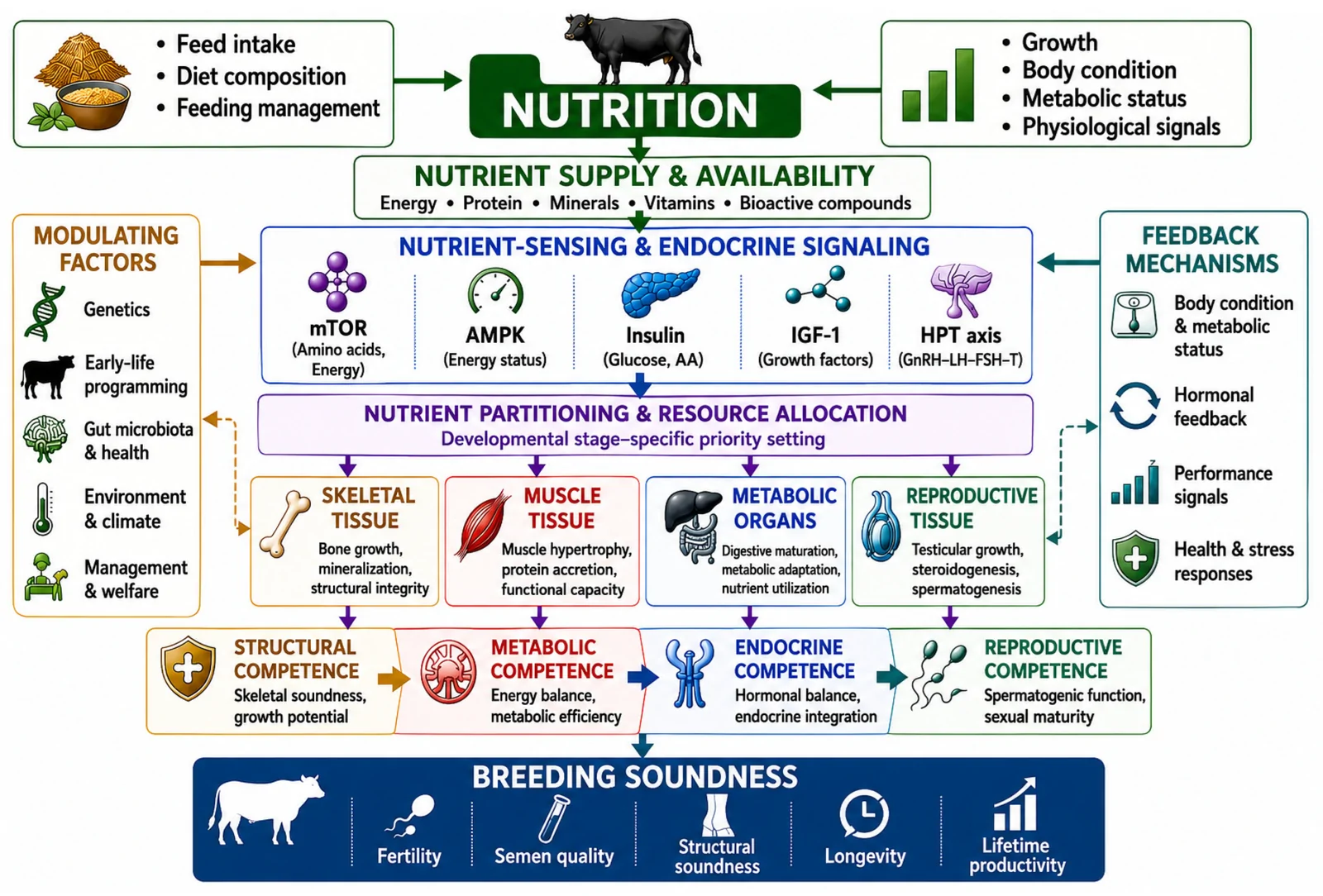
Conceptual framework of nutritional regulation during postnatal development in developing beef bulls. Nutrient availability activates nutrient-sensing and endocrine pathways that coordinate stage-specific nutrient partitioning among skeletal, muscular, metabolic, and reproductive tissues, driving the progressive establishment of structural, metabolic, endocrine, and reproductive competence that ultimately leads to breeding soundness.

Nutrition is a primary regulator of postnatal development in developing beef bulls, providing not only substrates for tissue growth but also metabolic and endocrine signals that coordinate cellular metabolism, tissue differentiation, physiological maturation, and reproductive development [7,17]. Through integrated nutrient-sensing pathways, nutrients regulate cellular proliferation, metabolic programming, developmental plasticity, and nutrient partitioning, thereby coordinating structural, metabolic, endocrine, and reproductive maturation [10,46,67]. Early-life nutritional interventions may induce persistent changes in endocrine responsiveness, metabolic function, and testicular development, highlighting the importance of developmental programming in determining future reproductive capacity [7,68]. This section examines how nutrition regulates postnatal development, with emphasis on developmental nutrient requirements, tissue maturation, reproductive development, and precision nutritional management (Table 3 and Figure 4).

### 3.1. Nutritional Regulation Through Nutrient-Sensing Pathways

Nutrition is a central regulator of postnatal development, functioning not only as a source of energy and structural substrates but also as a dynamic signaling system that coordinates tissue growth, metabolic adaptation, endocrine activity, and reproductive maturation [7,10]. During postnatal development, nutrients influence cellular behavior through evolutionarily conserved nutrient-sensing pathways that integrate nutritional status with developmental priorities. These signaling networks translate changes in nutrient availability into coordinated regulation of protein synthesis, cellular proliferation, differentiation, mitochondrial metabolism, and tissue remodeling, thereby linking nutritional inputs with long-term developmental outcomes [67,69,70].

Among the principal nutrient-sensing pathways, mechanistic target of rapamycin (mTOR) serves as a master regulator of anabolic metabolism by promoting protein synthesis, ribosome biogenesis, and cellular growth in response to amino acids, energy availability, insulin, and insulin-like growth factor-1 (IGF-1) [69,70,71]. In contrast, AMP-activated protein kinase (AMPK) functions as a cellular energy sensor that is activated during energy deficiency, promoting oxidative metabolism while suppressing energy-consuming anabolic processes [70,71]. Nutritional signals are further integrated through phosphatidylinositol 3-kinase (PI3K)–Akt signaling, which mediates many of the downstream effects of insulin and IGF-1 on cellular proliferation, nutrient utilization, and tissue growth. Together, these interconnected pathways coordinate metabolic flexibility by balancing anabolic and catabolic processes according to developmental stage and nutrient availability [67,69,70]. The biological consequences of nutrient sensing vary throughout postnatal development because tissues differ in their developmental priorities and metabolic responsiveness (Table 3). During the neonatal period, nutrient-sensitive pathways primarily support thermoregulation, passive immunity, gastrointestinal adaptation, and rapid organ development, with glucose and milk-derived lipids serving as the predominant energy sources [33,72]. As calves transition to solid feed, establishment of ruminal fermentation increases the availability of volatile fatty acids and microbial protein, leading to profound metabolic reprogramming that supports skeletal growth, muscle accretion, visceral organ maturation, and improved metabolic efficiency [33,61,62]. During the growing and peripubertal stages, nutrient partitioning progressively shifts toward lean tissue deposition, endocrine activation, testicular growth, and spermatogenesis while maintaining continued somatic growth [17,46]. Thus, developmental progression is governed not only by nutrient supply but also by stage-specific activation of nutrient-sensing pathways that prioritize nutrient allocation among competing physiological processes.

Developmental outcomes therefore depend on synchronizing nutrient supply with physiological demand rather than maximizing nutrient intake alone [46]. Nutritional restriction during critical developmental windows can suppress anabolic signaling, impair skeletal growth, delay metabolic maturation, and postpone reproductive development, whereas excessive energy intake may promote adipose deposition and metabolic dysregulation, with recent evidence in young beef bulls indicating potential adverse effects on subsequent semen quality [58,73]. Likewise, adequate supplies of essential amino acids, minerals, and vitamins are required to support bone mineralization, antioxidant defense, immune competence, mitochondrial function, and normal endocrine and reproductive development [74,75,76].

Collectively, these findings demonstrate that nutritional regulation during postnatal development extends far beyond meeting dietary requirements. Through coordinated activation of nutrient-sensing pathways, nutrition integrates metabolic, endocrine, and developmental signals to regulate tissue-specific growth, developmental programming, and nutrient partitioning according to physiological stage. This integrated regulatory framework provides the mechanistic basis for understanding how nutrition directs tissue maturation, reproductive development, and ultimately breeding soundness in developing beef bulls.

### 3.2. Nutritional Regulation of Tissue Development and Reproductive Maturation

Activation of nutrient-sensing pathways translates nutritional inputs into tissue-specific developmental responses because individual tissues differ in their developmental timing, metabolic activity, and responsiveness to nutritional and endocrine signals. Consequently, identical nutritional inputs may produce distinct biological outcomes depending on developmental stage, resulting in dynamic nutrient partitioning that supports the coordinated establishment of structural, metabolic, endocrine, and reproductive competence [7,33,77]. Rather than promoting uniform growth, nutritional regulation continuously prioritizes nutrient allocation to tissues with the greatest physiological demand, thereby synchronizing tissue maturation with changing developmental priorities (Figure 4).

During early postnatal development, nutritional signals primarily support skeletal growth and lean tissue accretion, establishing the structural framework required for subsequent metabolic and reproductive maturation [66,78]. Adequate energy and protein intake, together with balanced mineral and vitamin nutrition, promote bone mineralization, muscle protein synthesis, connective tissue development, and satellite cell activity, thereby supporting structural competence and long-term growth potential [74,76]. In contrast, nutritional restriction during these critical developmental windows suppresses anabolic processes and delays structural maturation, whereas excessive energy intake preferentially promotes adipose tissue deposition without proportional improvements in skeletal development or functional capacity [28,56,75].

As postnatal development progresses, nutrient partitioning gradually shifts from structural growth toward metabolic adaptation and reproductive maturation. Functional development of the rumen increases the availability of volatile fatty acids and microbial protein, improving nutrient utilization and metabolic efficiency while supporting endocrine activation and tissue-specific nutrient allocation [47,61,62]. These metabolic adaptations create the physiological conditions required for rapid testicular growth, differentiation of Sertoli and Leydig cells, and initiation of spermatogenesis during the peripubertal period [46,65]. Consequently, reproductive maturation depends not only on nutrient availability but also on the coordinated integration of metabolic adaptation, endocrine signaling, and tissue-specific developmental programming.

Collectively, tissue maturation reflects the coordinated interaction of nutrient sensing, metabolic regulation, endocrine signaling, and developmental programming rather than the independent growth of individual organs. By continuously adjusting nutrient partitioning according to developmental priorities, nutritional regulation ensures the progressive establishment of structural integrity, metabolic competence, endocrine function, and reproductive capacity required for breeding soundness [33,47,67].

### 3.3. Nutritional Regulation of Reproductive Maturation

Successful reproductive maturation depends on the coordinated interaction between nutritional status, metabolic regulation, and endocrine signaling throughout postnatal development. As structural growth progresses and metabolic competence is established, nutrient availability increasingly supports the development of the HPG axis, enabling the endocrine activation required for puberty and subsequent reproductive function [17,46,69]. Rather than acting independently, nutritional and endocrine signals operate as an integrated regulatory network that synchronizes somatic growth with reproductive maturation.

Adequate nutrition during the pre-weaning and growing periods establishes the metabolic conditions necessary for normal activation of the HPG axis during the peripubertal stage [33,47]. Improvements in nutrient utilization associated with rumen maturation enhance energy availability and metabolic efficiency, facilitating endocrine activation, increasing gonadotropin secretion, and promoting testicular growth [47,61,62]. These developmental transitions support the proliferation and maturation of Sertoli cells, differentiation of Leydig cells, and initiation of spermatogenesis, thereby determining future sperm-producing capacity and breeding potential [46,65,73]. Because Sertoli cell number is established largely before or around puberty, nutritional management during early postnatal development may have lasting consequences for adult reproductive capacity [65]. Nutritional imbalance during critical developmental windows may disrupt reproductive maturation through alterations in metabolic and endocrine regulation rather than through reduced nutrient supply alone. Prolonged nutritional restriction delays puberty, suppresses testicular development, and reduces spermatogenic capacity, whereas excessive energy intake may increase adiposity and impair metabolic homeostasis without proportionally improving reproductive development [28,46,56,71]. These findings emphasize that optimal reproductive development depends on balanced nutrient supply that supports coordinated metabolic adaptation and endocrine function rather than accelerated body weight gain alone.

Collectively, reproductive maturation represents the functional outcome of integrated nutritional regulation throughout postnatal development. By coordinating nutrient sensing, metabolic adaptation, endocrine activation, and testicular development, nutrition establishes the physiological foundation required for normal puberty, semen production, and breeding soundness. The endocrine mechanisms underlying these developmental processes are discussed in detail in Section 5.

## 4. Metabolic Regulation of Growth and Development in Developing Beef Bulls

**Figure 5:**
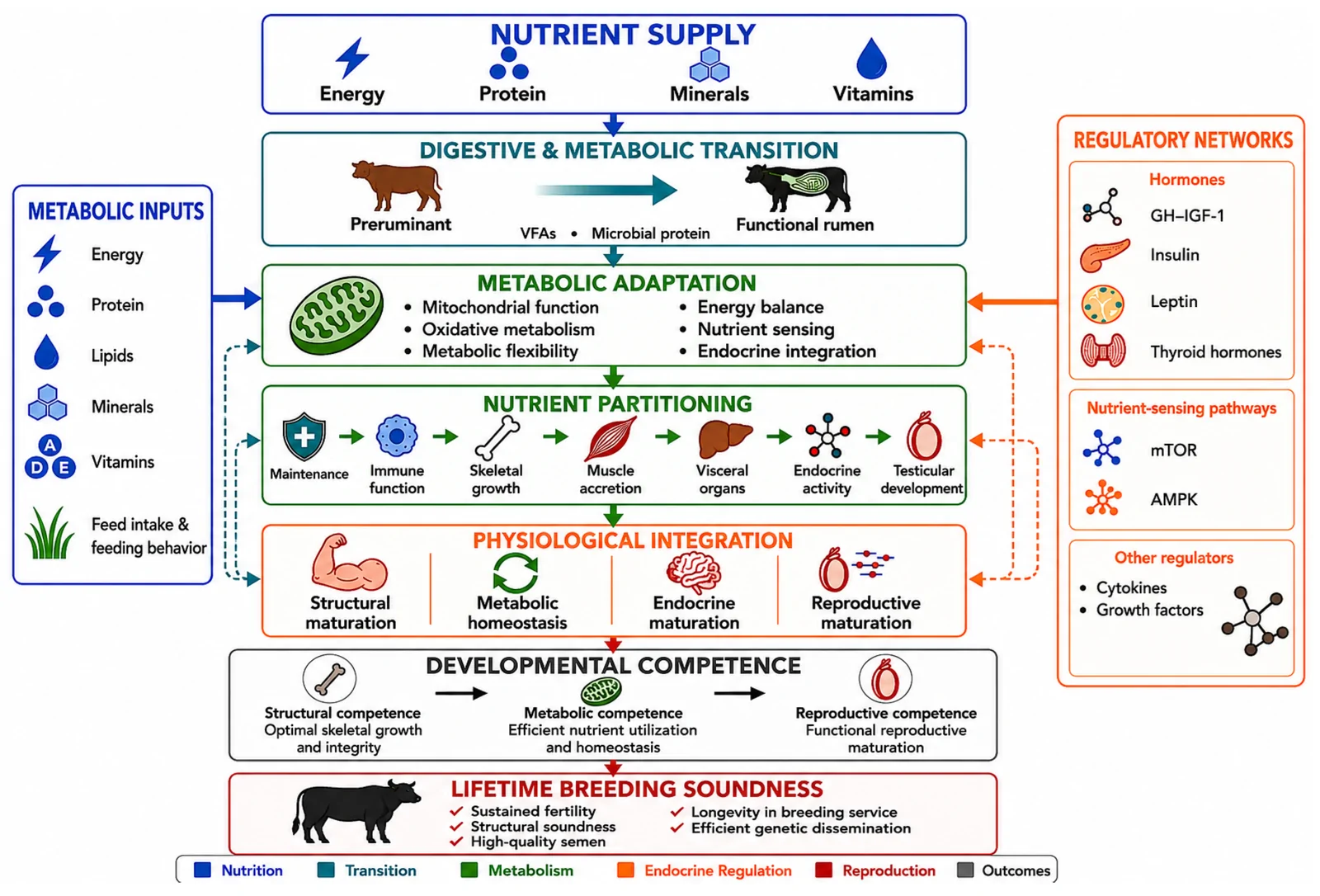
Integrated metabolic regulation of postnatal development in developing beef bulls. Nutrient supply and metabolic adaptation coordinate stage-specific nutrient partitioning through interactions with nutrient-sensing and endocrine pathways. These integrated processes support structural growth, metabolic homeostasis, endocrine maturation, and reproductive development, leading to developmental competence and ultimately breeding soundness.

Metabolism provides the physiological interface between nutrition and postnatal development by regulating nutrient utilization, partitioning, energy production, and their conversion into tissue maturation, physiological function, and reproductive capacity [10,33]. Throughout postnatal development, metabolic processes continuously adapt to changing physiological priorities through coordinated nutrient-sensing and endocrine signaling pathways, thereby regulating the establishment of structural, metabolic, endocrine, and reproductive competence [7,71]. Consequently, metabolic regulation influences not only developmental efficiency but also pubertal maturation, reproductive function, and ultimately breeding soundness. This section reviews the metabolic mechanisms underlying postnatal development, with emphasis on metabolic adaptation, nutrient partitioning, anabolic metabolism, metabolic biomarkers, and developmental competence (Figure 5).

### 4.1. Metabolic Adaptation and Nutrient Partitioning

Postnatal development is accompanied by continuous metabolic adaptation that enables developing beef bulls to meet the changing physiological demands of tissue maturation, metabolic homeostasis, and reproductive development. Rather than remaining static, metabolic processes adjust dynamically throughout development, optimizing nutrient utilization and coordinating the progressive establishment of structural, metabolic, endocrine, and reproductive competence [28,79]. These adaptive responses are mediated through coordinated changes in substrate utilization, mitochondrial energy production, nutrient sensing, and endocrine regulation, allowing developing bulls to match metabolic capacity with changing developmental priorities [46,80]. Central to these adaptations is nutrient partitioning, whereby metabolic resources are preferentially allocated among tissues according to stage-specific physiological demands rather than distributed uniformly throughout the body [12,81] (Figure 5).

One of the most important metabolic transitions during postnatal development is the shift from preruminant to functional ruminant metabolism. During early life, glucose and milk-derived lipids constitute the principal energy substrates supporting maintenance, thermoregulation, gastrointestinal maturation, and visceral organ development [64,82]. As solid feed intake increases, ruminal microbial fermentation progressively replaces glucose as the primary source of metabolic energy through the production of volatile fatty acids (VFAs). Acetate, propionate, and butyrate collectively support oxidative metabolism, hepatic gluconeogenesis, rumen epithelial development, and metabolic efficiency, thereby establishing the metabolic foundation for subsequent tissue maturation and physiological development [5,61,62]. Among these VFAs, butyrate is particularly important for stimulating rumen epithelial proliferation and differentiation, whereas propionate serves as the principal gluconeogenic precursor supporting hepatic glucose production and systemic glucose homeostasis [5,61,62].

As development progresses, nutrient partitioning shifts in response to changing physiological priorities. During early postnatal life, metabolic resources are directed primarily toward maintenance, immune function, and skeletal development, whereas later developmental stages are characterized by increasing allocation to skeletal muscle accretion, metabolic maturation, and reproductive development [28,33]. This capacity to continuously adjust substrate utilization according to developmental stage and nutrient availability improves metabolic efficiency and supports coordinated postnatal development [80,81].

Energy balance is a major determinant of developmental outcomes because it regulates nutrient partitioning, anabolic metabolism, and metabolic efficiency. Moderate positive energy balance promotes lean tissue accretion and normal developmental progression, whereas prolonged negative energy balance redirects metabolic resources toward maintenance and survival at the expense of skeletal development, muscle accretion, and reproductive maturation [79,80]. Conversely, excessive energy intake shifts nutrient partitioning toward adipose tissue deposition rather than lean tissue accretion, reducing metabolic efficiency and potentially compromising structural soundness, testicular development, and subsequent breeding performance [10,83]. Collectively, these metabolic adaptations ensure efficient utilization of nutrient resources to support coordinated postnatal development and establish the physiological foundation for subsequent endocrine regulation.

### 4.2. Protein and Lipid Metabolism During Development

Protein and lipid metabolism are fundamental regulators of postnatal development because they coordinate nutrient partitioning, lean tissue accretion, body composition, metabolic homeostasis, and reproductive maturation throughout development. In developing beef bulls, the balance between anabolic protein metabolism and lipid utilization determines not only developmental efficiency but also the establishment of structural, metabolic, endocrine, and reproductive competence [84,85]. Consequently, successful postnatal development depends not only on nutrient availability but also on the efficiency with which protein and lipid metabolism adapt to changing physiological priorities. Representative circulating metabolic biomarkers reported during successive developmental stages in healthy beef cattle are summarized in Table 4.

Protein metabolism is characterized by continuous turnover, in which protein synthesis and degradation are tightly coordinated to support tissue growth, remodeling, and physiological adaptation. During the pre-weaning and growing phases, net protein accretion predominates, promoting skeletal muscle hypertrophy, bone matrix formation, and development of the visceral and reproductive organs [85]. In ruminants, microbial protein synthesized within the rumen provides the principal source of absorbable amino acids, making efficient rumen function essential for lean tissue accretion and postnatal development [86]. Beyond supporting structural development, amino acids contribute to nitrogen metabolism, antioxidant defense, cellular signaling, and endocrine regulation, thereby facilitating physiological adaptation throughout postnatal development [85,87]. Sustained protein accretion is also essential for testicular growth, Sertoli and Leydig cell maturation, and spermatogenic development during the peripubertal period, directly linking protein metabolism with reproductive maturation [73].

**Table 4:** Representative circulating metabolic biomarkers during postnatal development of healthy beef cattle.

Biomarker	Neonatal/ Early Suckling	Pre- Weaning	Growing	Peripubertal/ Early Fattening	Biological Significance	References
Glucose (mg/dL)	109–134	95–110	73–95	66–72	Energy availability and metabolic adaptation	[77,88,89,90,91]
BHBA (mmol/L)	0.08–0.10	0.15–0.28	0.28–0.40	0.43–0.50	Rumen development and butyrate metabolism	[40,77,88]
NEFA (mmol/L)	0.24	0.18–0.22	0.17–0.18	0.15–0.18	Energy balance and lipid mobilization	[40,77,91,92]
BUN (mg/dL)	11.0	10.7–11.0	11.3	13.9	Protein metabolism and nitrogen utilization	[40,77,91,92]
Albumin (g/dL)	3.08	3.08–3.29	3.29–3.45	3.62	Protein status and hepatic function	[40,77,91,92]
Total cholesterol (mg/dL)	75–95	95–110	85–95	66–72	Lipid metabolism and steroidogenesis	[77,89,91]
Creatinine (mg/dL)	0.82	0.82–0.94	0.94–1.06	1.18	Muscle development and renal function	[77,92]

Lipid metabolism complements protein accretion by regulating energy storage, fatty acid oxidation, substrate utilization, and body composition. During early postnatal life, milk-derived lipids serve as the principal metabolic fuels supporting maintenance, thermoregulation, and organ development, whereas advancing postnatal development is accompanied by progressive adipose tissue deposition as lean tissue accretion gradually declines [28,93]. Appropriate lipid metabolism maintains metabolic flexibility by balancing lipid oxidation with storage, enabling developing bulls to adapt efficiently to fluctuations in nutrient availability.

Although moderate adipose reserves contribute to energy homeostasis, excessive adipose tissue deposition may be associated with less favorable nutrient partitioning and reduced lean tissue accretion, potentially compromising developmental efficiency and subsequent breeding soundness [81,93]. Accordingly, nutritional management should promote lean tissue accretion while preventing excessive adiposity that compromises structural soundness and breeding potential.

### 4.3. Metabolic Biomarkers of Growth and Development

Metabolic biomarkers provide dynamic indicators of physiological status and have become valuable tools for evaluating postnatal development, nutritional adequacy, metabolic adaptation, and reproductive maturation in developing beef bulls. Unlike conventional growth traits, circulating metabolites reflect ongoing physiological processes and can provide complementary information on nutritional and metabolic status that may not be captured by measurements of body weight or body composition alone [28,94,95]. Consequently, metabolic profiling provides an objective means of assessing developmental competence and monitoring the effectiveness of nutritional and management strategies throughout postnatal development.

Energy-related biomarkers are widely used to evaluate metabolic adaptation during postnatal development. Blood glucose reflects energy availability, particularly during early postnatal life, whereas β-hydroxybutyrate (BHBA) increases as rumen fermentation becomes established and serves as a functional biomarker of rumen development, ketogenesis, and metabolic adaptation [30,33,40]. Non-esterified fatty acids (NEFA) provide important indicators of lipid mobilization and whole-body energy balance, with elevated circulating concentrations generally reflecting increased mobilization of adipose tissue reserves during periods of negative energy balance, nutritional restriction, or metabolic stress [96,97].

Protein metabolism can be evaluated using circulating biomarkers such as blood urea nitrogen (BUN), total protein, albumin, and creatinine. BUN reflects nitrogen utilization and the balance between dietary protein supply and protein metabolism, whereas total protein and albumin provide indicators of protein nutritional status, hepatic protein synthesis, and overall metabolic status [33,98]. Creatinine is closely associated with skeletal muscle mass and muscle protein turnover and may serve as an indirect indicator of lean tissue development during growth. Interpretation of metabolic biomarkers should always consider developmental stage, nutritional status, and physiological maturity, because circulating metabolite concentrations and reference intervals change continuously throughout postnatal development [98,99].

Recent advances in metabolomics have expanded the application of metabolic biomarkers beyond conventional blood biochemistry. High-throughput metabolomic profiling enables simultaneous characterization of metabolites involved in energy, protein, lipid, and amino acid metabolism, providing a more comprehensive assessment of metabolic adaptation, developmental physiology, and physiological status [94,95,100]. Although these approaches remain primarily research tools, they show considerable promise for identifying biomarkers associated with developmental efficiency, metabolic resilience, reproductive maturation, and breeding potential. Integration of metabolomic data with phenotypic, endocrine, and genomic information may improve the precision of nutritional and genetic management while facilitating the early identification of superior replacement sires [100,101].

### 4.4. Metabolic Efficiency as a Determinant of Developmental Competence

Metabolic efficiency represents the integrated outcome of nutrient utilization, metabolic adaptation, and physiological regulation throughout postnatal development. Rather than simply reflecting feed conversion or growth rate, metabolic efficiency reflects the capacity of the animal to partition nutrients appropriately among structural growth, tissue maintenance, endocrine activity, immune function, and reproductive development according to developmental stage and physiological demands [81,102]. Consequently, metabolic efficiency is a key determinant of developmental competence and successful progression toward breeding soundness. In total, metabolic regulation links nutritional inputs with endocrine signaling and tissue maturation, providing the physiological foundation for the endocrine mechanisms governing postnatal development discussed in the following section.

Recent advances in systems biology have demonstrated that metabolic efficiency arises from the coordinated interaction of nutrient partitioning, mitochondrial function, oxidative metabolism, and whole-body physiological regulation rather than from individual metabolic pathways acting in isolation [81,102]. Integrating metabolic biomarkers with phenotypic, endocrine, and emerging omics data therefore provides a more comprehensive assessment of developmental status and may facilitate earlier identification of bulls with superior growth efficiency and reproductive potential.

## 5. Endocrine Regulation of Growth and Reproductive Development in Developing Beef Bulls

**Figure 6:**
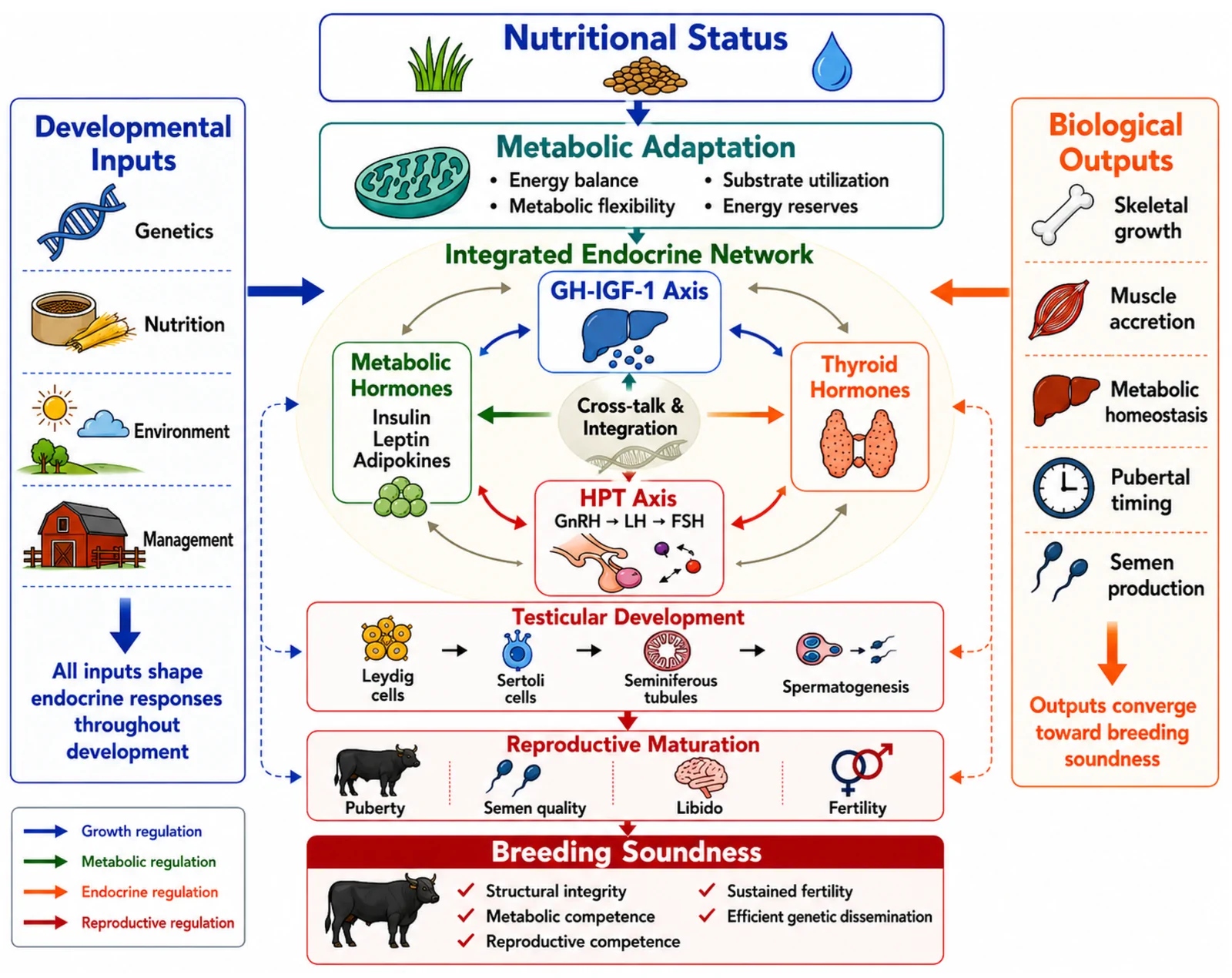
Integrated endocrine regulation of postnatal development in developing beef bulls. Endocrine regulation integrates nutritional status, metabolic adaptation, genetic potential, environmental influences, and management through coordinated interactions among the GH–IGF-1 axis, metabolic hormones, thyroid hormones, and the hypothalamic–pituitary–gonadal (HPG) axis. These endocrine networks coordinate tissue maturation, metabolic homeostasis, pubertal development, testicular maturation, and reproductive function, progressively establishing structural, metabolic, endocrine, and reproductive competence that ultimately leads to breeding soundness.

Endocrine regulation coordinates the developmental processes initiated by nutrition and metabolism, ensuring that tissue maturation, metabolic adaptation, and reproductive development proceed in a synchronized manner throughout postnatal life. Rather than functioning independently, endocrine networks integrate nutritional status, metabolic activity, genetic potential, and environmental cues to regulate cell proliferation, tissue differentiation, nutrient partitioning, and functional maturation, thereby coordinating the progressive establishment of structural, metabolic, endocrine, and reproductive competence [7,10,46]. As postnatal development progresses, endocrine signaling directs the transition from predominantly somatic development to reproductive competence through coordinated interactions among the growth hormone–insulin-like growth factor-1 (GH–IGF-1) axis, metabolic hormones, thyroid hormones, and the HPG axis [45,68]. Consequently, breeding soundness depends not only on adequate nutrition and metabolic adaptation but also on the precise temporal regulation of endocrine networks that coordinate structural development, pubertal maturation, and reproductive function [11,60]. This section reviews the endocrine mechanisms regulating postnatal development, with emphasis on hormonal regulation of tissue growth, metabolic adaptation, and maturation of the HPG axis (Figure 6).

### 5.1. Growth and Metabolic Endocrine Regulation

Postnatal development is regulated by an integrated endocrine network that coordinates nutrient availability with tissue maturation and metabolic adaptation. Rather than acting independently, growth hormone (GH), insulin-like growth factor-1 (IGF-1), insulin, thyroid hormones, and adipokines interact to synchronize skeletal development, muscle accretion, nutrient partitioning, and metabolic homeostasis according to developmental stage [103,104]. This coordinated endocrine regulation enables developing beef bulls to progressively establish structural and metabolic competence before achieving full reproductive maturity [11,105]. The GH–IGF-1 axis is the principal endocrine regulator of postnatal somatic development. Growth hormone stimulates hepatic and local production of IGF-1, which promotes longitudinal bone growth, skeletal muscle hypertrophy, protein synthesis, and tissue remodeling throughout postnatal development [28,104]. During the growing phase, GH–IGF-1 signaling contributes to lean tissue accretion and coordinates nutrient utilization with changing developmental demands [106]. However, the relationship between GH and IGF-1 is strongly influenced by nutritional status. In bull calves, reduced nutrient intake can alter somatotropic-axis development and decrease tissue responsiveness to GH, resulting in lower circulating IGF-1 despite maintained or elevated GH concentrations [107].

Insulin and thyroid hormones complement GH–IGF-1 signaling by regulating energy metabolism, nutrient utilization, and cellular growth [104]. Insulin promotes glucose uptake, amino acid utilization, and anabolic metabolism, whereas thyroid hormones regulate basal metabolic rate, mitochondrial activity, and tissue differentiation [85]. Together, these hormones ensure efficient nutrient partitioning to support structural development while maintaining metabolic homeostasis throughout postnatal development [108]. In addition, adipokines, particularly leptin, provide endocrine signals reflecting body energy reserves and contribute to the regulation of metabolic status, developmental progression, and reproductive maturation [108].

Recent evidence indicates that endocrine regulation is governed by coordinated interactions among growth-, metabolic-, and reproductive-related hormonal pathways rather than by the actions of individual hormones in isolation. Cross-talk among the GH–IGF-1 axis, metabolic hormones, and the HPG axis enables developing bulls to adjust nutrient partitioning, tissue maturation, and developmental progression in response to nutritional status and physiological demands [7,43,104]. Consequently, endocrine regulation should be regarded as an integrated biological network that links nutritional inputs with postnatal development, providing the physiological foundation for activation of the reproductive axis and subsequent pubertal maturation (Figure 6).

### 5.2. Hypothalamic–Pituitary–Gonadal Axis and Pubertal Development

The transition from somatic development to reproductive maturity is governed by activation of the HPG axis, which integrates neuroendocrine signaling with progressive maturation of the male reproductive system [17]. Gonadotropins regulate testicular maturation by establishing the endocrine and cellular environment required for spermatogenesis, steroidogenesis, and ultimately reproductive competence [7,10,17]. Consequently, puberty should be regarded as a coordinated developmental transition rather than a single physiological event.

Within the testes, luteinizing hormone (LH) stimulates Leydig cells to produce testosterone, promoting testicular growth, accessory sex gland development, secondary sexual characteristics, and maintenance of spermatogenesis [10,46,109]. Follicle-stimulating hormone (FSH) acts primarily on Sertoli cells to promote germ-cell differentiation, seminiferous tubule maturation, and spermatogenesis [109]. The coordinated actions of Sertoli and Leydig cells progressively establish the structural and functional capacity required for normal fertility [11]. Although the onset of spermatogenesis marks the beginning of reproductive capability, continued post-pubertal maturation is required to achieve optimal semen quality, sperm output, and breeding performance [47,110].

Activation of the HPG axis is influenced by genotype, nutritional status, developmental trajectory, metabolic status, and environmental conditions [43].

Adequate nutrition and metabolic competence promote endocrine maturation and testicular development, whereas nutritional restriction, disease, or chronic stress suppress gonadotropin secretion, delay puberty, and impair reproductive development [10]. These coordinated endocrine and metabolic interactions ensure that reproductive maturation proceeds only after adequate structural and metabolic competence has been established, thereby synchronizing fertility with overall developmental readiness [11,111].

Current evidence indicates that reproductive competence extends beyond the onset of puberty. Continued post-pubertal maturation improves semen concentration, motility, morphology, libido, and mating behavior, thereby enhancing fertility and the capacity for sustained natural service [3,5]. Therefore, successful development of developing beef bulls depends not only on timely activation of the HPG axis but also on progressive maturation of reproductive function that ultimately determines breeding soundness and lifetime reproductive performance.

### 5.3. Endocrine Coordination of Reproductive Competence

Endocrine regulation of postnatal development is achieved through coordinated interactions among growth-, metabolic-, and reproduction-related hormonal pathways rather than through the actions of individual hormones in isolation [104]. Throughout development, endocrine networks integrate nutritional status, metabolic activity, genetic potential, and environmental cues to synchronize tissue maturation, metabolic adaptation, and reproductive development according to stage-specific physiological priorities [5,43]. This integrated regulation ensures that reproductive maturation proceeds only after adequate structural and metabolic competence has been established.

Recent evidence indicates that endocrine signaling is highly dynamic, involving continuous cross-talk among the GH–IGF-1 axis, metabolic hormones, thyroid hormones, and the HPG axis [17,104]. These interactions coordinate nutrient utilization, tissue differentiation, pubertal development, and reproductive function while maintaining physiological homeostasis [104]. Consequently, disturbances in endocrine regulation arising from nutritional imbalance, metabolic stress, disease, or environmental challenges during critical developmental windows may alter developmental trajectories, delay puberty, and compromise fertility. Collectively, endocrine regulation integrates nutritional, metabolic, and physiological signals to coordinate postnatal development, providing the mechanistic link between developmental competence and the practical assessment of breeding potential discussed in the following section.

## 6. Assessment of Developmental Progress and Breeding Potential in Prospective Breeding Bulls

**Figure 7:**
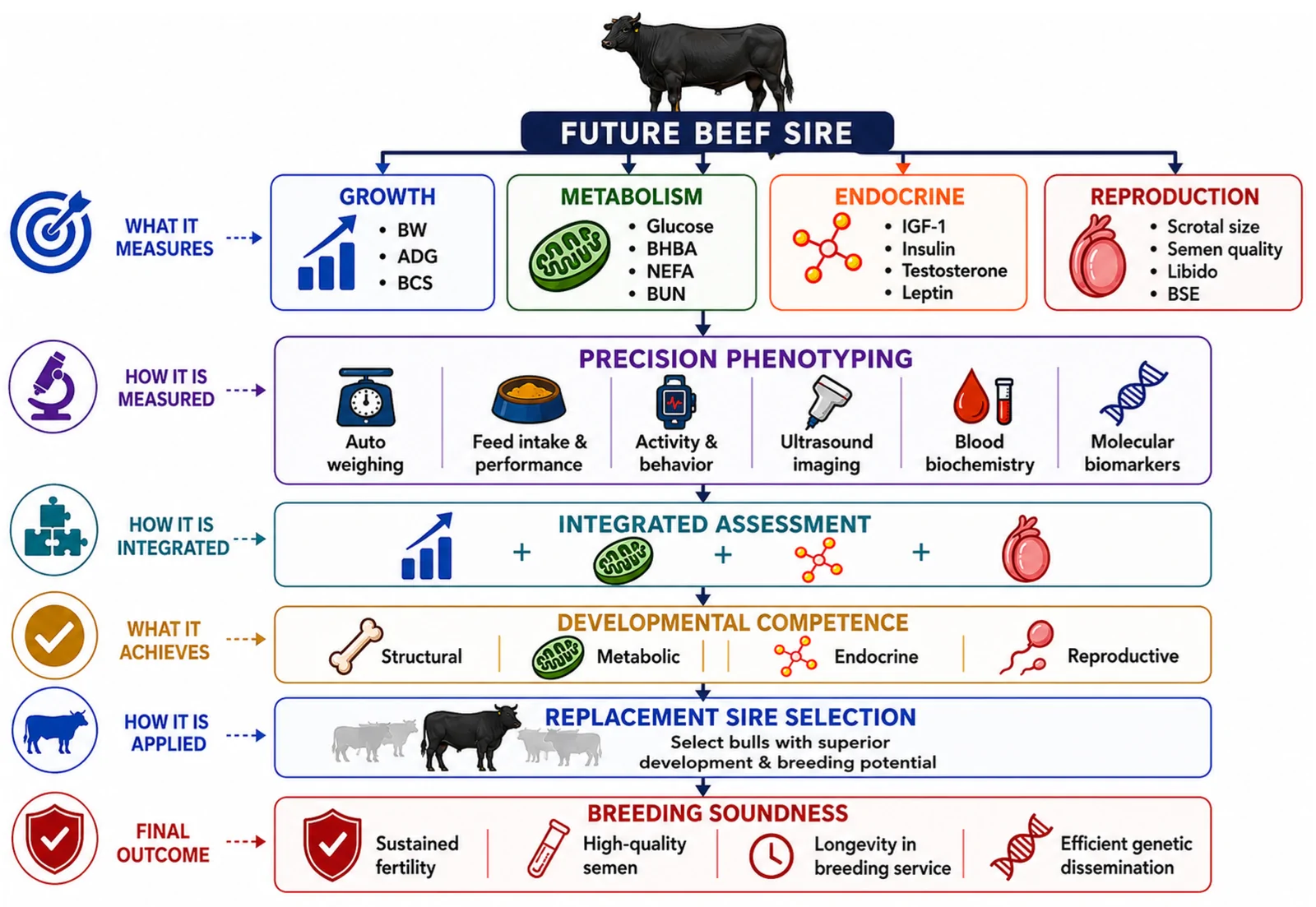
Integrated assessment of developmental competence in prospective breeding bulls. Developmental competence is evaluated through the integration of structural, metabolic, endocrine, reproductive, and precision phenotyping indicators. This systems-based assessment improves prediction of breeding potential, replacement sire selection, and breeding soundness.

Successful postnatal development in developing beef bulls is ultimately determined by the coordinated establishment of structural, metabolic, endocrine, and reproductive competence rather than by growth performance alone [45]. Consequently, assessment of developmental progress requires an integrated evaluation of phenotypic, physiological, metabolic, endocrine, and reproductive characteristics that collectively reflect developmental status and future breeding potential [60]. Although traditional measures such as body weight and average daily gain remain valuable indicators of somatic development, they provide only a partial assessment of biological maturity and future reproductive performance [3]. Contemporary approaches increasingly integrate structural phenotyping, metabolic and endocrine biomarkers, reproductive assessments, and precision phenotyping to identify bulls with superior developmental competence and long-term breeding potential [5,108,112]. This section reviews the principal approaches for assessing postnatal development and breeding readiness in prospective breeding bulls (Figure 7).

### 6.1. Growth and Structural Indicators of Development

Growth and structural traits provide the first objective assessment of developmental progress because they reflect the cumulative effects of genetic potential, nutrition, metabolism, endocrine regulation, and overall health throughout postnatal development [113,114]. Although body weight and average daily gain remain widely used indicators of growth, these measures alone do not adequately characterize developmental competence because animals with similar growth rates may differ substantially in skeletal maturity, body composition, metabolic status, and reproductive potential [112,113]. Consequently, growth measurements should be interpreted alongside structural indicators to provide a more comprehensive assessment of future breeding performance.

Structural measurements, including withers height, hip height, body length, heart girth, body depth, and pelvic dimensions, provide valuable information on skeletal development, frame size, and overall structural maturity [115,116]. These traits reflect establishment of the musculoskeletal framework that underpins locomotion, mating ability, structural soundness, and sustained reproductive performance under natural breeding conditions [44,113]. Likewise, body condition score (BCS) complements growth measurements by assessing body energy reserves and nutritional status, helping distinguish appropriate lean tissue development from excessive or inadequate adiposity [112,113,117]. Maintaining optimal body condition is particularly important because both inadequate body reserves and excessive adiposity impair locomotion, metabolic efficiency, breeding soundness, and subsequent reproductive performance [118,119].

Developmental trajectory is increasingly recognized as a more informative indicator of developmental progress than body weight measured at a single time point. Longitudinal assessment of body weight, skeletal dimensions, and body condition enables evaluation of developmental consistency while facilitating early identification of animals exhibiting suboptimal developmental trajectories, disproportionate tissue maturation, or delayed physiological development [66,78]. Longitudinal phenotyping provides the foundation for precision developmental management by enabling earlier prediction of developmental competence than conventional end-point measurements. This approach is particularly valuable during the pre-weaning, growing, and peripubertal periods, when developmental trajectories remain highly responsive to nutritional and management interventions [79,112].

### 6.2. Reproductive Indicators and Breeding Soundness Evaluation

Reproductive indicators provide the most direct assessment of breeding potential because they reflect the functional maturation of the male reproductive system [5,45]. Unlike growth and structural traits, which primarily characterize somatic development, reproductive indicators evaluate the establishment of endocrine function, testicular maturation, spermatogenesis, and fertility [5,60]. Consequently, assessment of breeding potential requires integration of reproductive measurements with structural, metabolic, and endocrine indicators to determine whether a bull has attained functional reproductive competence [3,5].

Scrotal circumference is one of the most informative and widely used indicators of reproductive development because it is closely associated with testicular size, sperm-producing capacity, and age at puberty [10,114]. As testicular development progresses throughout the peripubertal period, increasing scrotal circumference generally reflects expansion of the seminiferous tubules, maturation of Sertoli and Leydig cell function, and progressive establishment of spermatogenesis [17,114]. Because of its strong association with daily sperm production, semen quality, and fertility potential, scrotal circumference is routinely incorporated into breeding soundness evaluations and replacement sire selection programs [52,120].

Although puberty marks the onset of sperm production, reproductive competence continues to improve throughout the post-pubertal period [11,83]. Progressive increases in sperm concentration, motility, morphology, and overall ejaculate quality accompany continued testicular maturation, while libido, sexual behavior, and mating ability also continue to develop [11,83]. Consequently, evaluation of semen quality provides essential information on functional reproductive capacity beyond the timing of puberty alone [45,60].

Breeding soundness is best assessed through an integrated evaluation combining physical examination, reproductive tract assessment, and semen quality analysis [60]. This comprehensive approach recognizes that fertility is a multifactorial trait resulting from the coordinated establishment of structural soundness, endocrine function, testicular maturation, spermatogenesis, and sperm functional competence rather than any single reproductive characteristic [5,121]. Consequently, breeding soundness represents the practical culmination of successful postnatal development and integrates the biological processes discussed throughout this review into an objective assessment of future reproductive performance.

### 6.3. Emerging Biomarkers and Precision Phenotyping

Assessment of prospective breeding bulls is evolving from conventional phenotypic evaluation toward integrated approaches combining physiological, molecular, and digital phenotyping [122,123]. Although growth traits, body measurements, scrotal circumference, and semen quality remain fundamental indicators of developmental progress, advances in precision livestock technologies enable developmental competence to be evaluated with greater accuracy, higher throughput, and at earlier stages of life [122,124].

Metabolic and endocrine biomarkers provide dynamic information on nutritional status, metabolic adaptation, tissue maturation, and reproductive development and may complement conventional phenotypic measurements in breeding bull evaluation [5,123]. Importantly, their role as indicators of current physiological status should be distinguished from validated prediction of subsequent reproductive performance. Nevertheless, longitudinal evidence in Japanese Black beef bulls has shown that prepubertal concentrations of IGF-I, INSL3, and inhibin were associated with subsequent semen abnormalities, suggesting that selected endocrine biomarkers may have predictive potential when validated against later reproductive outcomes [125]. Similarly, advances in transcriptomics, metabolomics, proteomics, and other omics technologies have facilitated the identification of molecular signatures associated with developmental efficiency and reproductive traits [126,127]. Although these approaches are not yet routinely implemented in commercial breeding programs, they may provide complementary biological information for evaluating developmental trajectories and, following appropriate longitudinal validation, have the potential to contribute to earlier prediction of reproductive potential and breeding soundness.

Rapid advances in precision phenotyping have further expanded the capacity to monitor postnatal development in developing beef bulls [115,122]. Automated body measurements, imaging technologies, wearable sensors, and computer vision systems enable continuous assessment of growth, body condition, locomotion, and behavioral activity with minimal animal handling [124,128]. When integrated with genomic, physiological, endocrine, and reproductive data, these technologies enable more objective evaluation of developmental trajectories and facilitate data-driven selection of superior replacement sires [129,130].

## 7. Future Perspectives: From Precision Phenotyping to Predictive Development of Prospective Breeding Bulls

**Figure 8:**
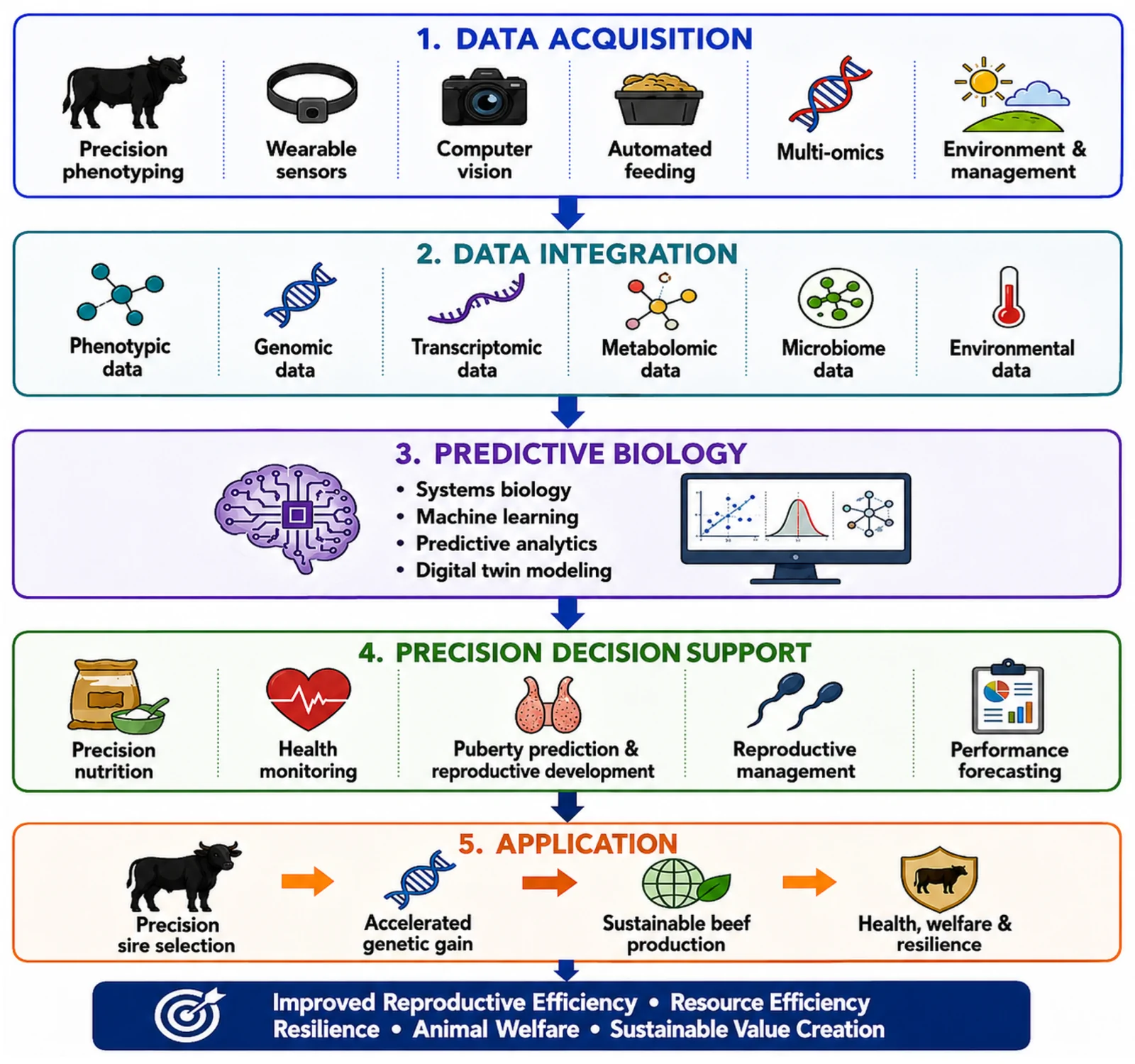
Future framework for precision development of prospective breeding bulls. Integration of precision phenotyping, multi-omics, systems biology, and artificial intelligence may support predictive biological frameworks that support precision nutritional management, early assessment of developmental competence, replacement sire selection, and sustainable genetic improvement.

The development of prospective breeding bulls is shifting from evaluating mature phenotypes to predicting developmental trajectories throughout postnatal life [79]. Although growth performance, structural soundness, and breeding soundness evaluation remain essential, they mainly assess animals after important developmental processes have already occurred [4,5]. Current evidence shows that postnatal development is regulated by continuous interactions among nutrition, metabolism, endocrine and immune function, tissue maturation, genetics, and environmental factors [8,14,27]. Future breeding programs will increasingly integrate these biological data to identify animals with superior breeding potential at earlier stages of development (Figure 8).

Systems biology provides the basis for this approach by integrating nutrition, metabolism, endocrine regulation, immune function, reproductive development, and environmental influences into a single biological framework [111,129]. Combined with precision livestock farming, automated phenotyping, wearable sensors, imaging technologies, multi-omics, and artificial intelligence, these approaches enable continuous monitoring of postnatal development and improve prediction of nutritional requirements, reproductive development, health, and breeding potential [115,122,128,131].

An important future direction is the development of predictive decision-support systems that combine phenotypic, physiological, molecular, reproductive, and environmental information to guide management throughout postnatal life [122,124,131,132]. Digital twin technologies may further improve this process by creating digital models of individual animals that predict growth, metabolic adaptation, and reproductive development [131,133,134]. However, wider application will require standardized phenotyping, validated biomarkers, shared biological databases, and reliable analytical methods [122,131,135].

Ultimately, future beef sire development will depend on integrating biological information from molecular, physiological, phenotypic, and environmental levels into predictive management systems. This integrated approach has the potential to improve reproductive efficiency, accelerate genetic gain, increase production efficiency, strengthen animal health and resilience, and support more sustainable beef production systems [129,133].

### 7.1. Precision Nutrition and Individualized Development

Nutritional management of developing beef bulls is increasingly being informed by approaches that account for individual variation in growth, body composition, metabolic status, and developmental stage. Precision nutrition technologies, including automated phenotyping, metabolic biomarkers, and sensor-derived measurements, may enable more individualized nutritional strategies than conventional population-based feeding programs [8,136,137]. Although feeding recommendations based on breed, age, body weight, and production stage remain useful, animals with similar genetics and nutrient intake often differ in developmental rate, metabolic adaptation, endocrine function, immune status, and reproductive maturation [36,102]. These differences highlight the need for nutritional management based on individual developmental trajectories.

Within a systems nutrition approach, nutrient requirements are determined by interactions among genetics, developmental stage, endocrine regulation, metabolic status, health, the rumen microbiome, and environmental conditions [138,139]. In addition to supporting tissue growth, nutrients also regulate cell proliferation, differentiation, nutrient partitioning, mitochondrial function, endocrine activity, and tissue maturation through nutrient-sensing pathways [28,53]. Therefore, precision nutrition aims to match nutrient supply with changing physiological priorities to support structural development, metabolic adaptation, immune function, and reproductive maturation.

Advances in precision livestock technologies may support individualized nutritional management through automated feeding systems and continuous monitoring of feed intake, body weight, body composition, activity, rumination, behavior, and physiological biomarkers [136,140]. Integration of these data with genomic information and longitudinal records enables early identification of animals with different developmental trajectories and supports timely nutritional intervention during critical stages of development [13,14,79,122,141] (Figure 8).

Artificial intelligence may further support precision nutrition by combining nutritional, physiological, genomic, environmental, and behavioral information to support adaptive nutritional management throughout postnatal development [124,131,142]. In the future, digital twin technologies may improve these approaches by creating digital models of individual animals that continuously update predictions of growth, metabolic adaptation, and reproductive development as new information becomes available [133,134]. Although these technologies are still developing, they have strong potential to improve developmental competence, reproductive efficiency, genetic gain, production efficiency, animal health, and the sustainability of beef production [131,137].

### 7.2. Precision Phenotyping, Multi-Omics, and Predictive Biology

Evaluation of prospective breeding bulls is shifting from conventional phenotype-based assessment toward integrated evaluation of postnatal development using high-dimensional biological data [122,131]. Traditionally, breeding decisions have relied on body weight, growth rate, structural traits, and breeding soundness evaluated near or after puberty [4]. Although these measurements remain essential, they mainly assess animals after important developmental processes have already occurred [5,123]. New approaches instead focus on continuous monitoring of postnatal development, allowing earlier identification of animals with superior developmental potential, physiological maturity, and reproductive performance [143,144].

Precision phenotyping plays a central role in this transition by enabling continuous and objective measurement of body growth, body composition, structural development, locomotion, feeding behavior, rumination, physiological status, and health using computer vision, three-dimensional imaging, wearable sensors, automated weighing systems, thermal imaging, and behavioral monitoring [124,145,146]. These longitudinal data improve the detection of individual differences in postnatal development compared with conventional periodic measurements.

Advances in genomics, transcriptomics, epigenomics, proteomics, metabolomics, microbiome research, and single-cell technologies are improving our understanding of the molecular mechanisms regulating nutrient utilization, tissue differentiation, metabolic adaptation, endocrine function, immune responses, and reproductive development [22,147,148]. Integrating these complementary datasets provides a more complete understanding of postnatal development than any single technology alone [95,147].

Artificial intelligence and machine learning may contribute to predictive biology by combining phenotypic, molecular, physiological, environmental, and management data to identify factors affecting developmental outcomes and support data-driven management decisions [131,135,143,149]. As biological datasets continue to expand, these models may provide increasingly informative predictions throughout postnatal development [142,150]. Digital twin technologies may further support this process by creating digital models of individual animals that predict responses to nutritional, environmental, and health interventions before they are implemented [133,134,151].

Ultimately, integrating precision phenotyping, multi-omics, systems biology, and artificial intelligence may improve prediction of postnatal development, enable earlier selection of superior replacement sires, enhance reproductive efficiency, accelerate genetic gain, and support sustainable beef production [129,131,133,147].

### 7.3. Knowledge Gaps and Future Research Priorities

Despite major advances in developmental physiology, nutrition, reproductive biology, and precision livestock technologies, important knowledge gaps still limit the application of predictive management in prospective breeding bulls [8,57,122]. Postnatal development is increasingly recognized as an integrated process regulated by interactions among genetics, nutrition, metabolism, endocrine and immune function, tissue maturation, and environmental factors [152,153]. Future research should therefore focus on integrating these biological processes across molecular, tissue, whole-animal, and environmental levels to improve understanding of postnatal development [147,152].

A major priority is the identification and validation of reliable biomarkers that predict reproductive potential early in life [123]. Although many metabolic, endocrine, transcriptomic, proteomic, and metabolomic biomarkers have been associated with growth, puberty, and fertility, few have shown consistent predictive value across breeds, production systems, and nutritional environments [123,154,155]. Large-scale longitudinal studies from birth to breeding age are, therefore, needed to identify reliable predictors of long-term reproductive performance [130,156].

Future progress may also depend on integrating longitudinal phenotyping with genomic, transcriptomic, metabolomic, endocrine, microbiome, behavioral, environmental, and management data into predictive biological models [131,147]. Artificial intelligence and machine learning may play an important role in this process, but future efforts should emphasize transparent models supported by standardized phenotyping, harmonized data collection, shared databases, and validation across different breeds and production systems [131,143,149,155,157].

Another important challenge is translating biological discoveries into practical breeding and management tools. Although many biomarkers and precision livestock technologies have shown promise under research conditions, further validation is required before routine commercial use [123,158,159]. Developing practical, cost-effective, and user-friendly decision-support systems may require collaboration among animal breeders, nutritionists, reproductive physiologists, veterinarians, engineers, and data scientists [150,160].

Ultimately, the development of prospective breeding bulls may depend on shifting from evaluation of mature phenotypes to prediction of postnatal development. Integrating systems biology, precision nutrition, longitudinal phenotyping, multi-omics, and artificial intelligence may enable earlier identification of superior replacement sires, improve reproductive efficiency, accelerate genetic gain, strengthen animal health and resilience, and support more sustainable beef production systems [129,142,143,148,153,161,162].

## 8. Conclusions

The successful development of prospective breeding bulls depends on the integrated regulation of nutrition, metabolism, endocrine function, tissue maturation, and reproductive development throughout postnatal life rather than on growth performance alone. These interacting biological processes progressively establish structural, metabolic, endocrine, and reproductive competence, ultimately determining breeding soundness and lifetime reproductive performance. Advances in metabolic and endocrine biomarkers, precision phenotyping, multi-omics, and artificial intelligence are shifting the evaluation of developing beef bulls from conventional assessment of mature phenotypes toward prediction of postnatal development. Integrating these approaches with systems biology may improve early identification of superior replacement sires, support more precise nutritional and management interventions, enhance reproductive efficiency and genetic gain, and promote more sustainable beef production. Although these approaches may ultimately enhance early prediction and precision management of prospective breeding bulls, their practical implementation requires validation across breeds, production systems, developmental stages, and independent populations. This review provides an integrated framework for understanding the biological regulation of postnatal development in developing beef bulls and highlights opportunities for future research and precision breeding strategies to optimize breeding potential and long-term reproductive performance.

## Figures and Tables

**Table 1:** Major physiological transitions during postnatal development of developing beef bulls.

Trait	Birth	Weaning	Growing	Puberty	Young Sire
Scrotal circumference	8–12 cm	16–20 cm	24–28 cm	28–34 cm	>34 cm
Testosterone	Low	Low	Increasing	Rapid increase	Mature
GH	High	High	Moderate	Moderate	Stable
IGF-1	Low	Increasing	High	Peak	Stable
Rumen papillae	Immature	Developing	Functional	Mature	Mature
Lean tissue accretion	Moderate	High	Highest	Moderate	Stable

**Table 2:** Tissue-specific maturation during postnatal development of developing beef bulls and its contribution to breeding soundness.

Tissue/System	Predominant Period of Maturation	Functional Contribution	Representative Indicators
Skeleton	Birth → Growing	Structural support, locomotor soundness, mating ability	Withers and hip height, skeletal dimensions, bone mineralization
Skeletal muscle	Pre-weaning → Puberty	Lean tissue accretion, locomotor capacity, metabolic function	Average daily gain, loin eye area, muscle hypertrophy
Adipose tissue	Growing → Post-puberty	Energy reserves, endocrine regulation, body condition	Body condition score, backfat thickness
Gastrointestinal tract	Birth → Weaning	Digestive maturation, nutrient utilization, metabolic adaptation	Rumen papillae development, feed intake, volatile fatty acid production
Endocrine system	Growing → Puberty	Coordination of growth, metabolism, and reproductive maturation	GH, IGF-1, insulin, testosterone
Male reproductive system	Peripubertal → Young sire	Spermatogenesis, fertility, breeding soundness	Scrotal circumference, testicular volume, semen quality

**Table 3:** Stage-specific developmental priorities, nutritional sensitivity, and potential long-term consequences of inadequate nutrition during postnatal development in developing beef bulls.

Developmental Window	Major Developmental Event	Nutritional Sensitivity	Potential Long-Term Consequence of Inadequate Nutrition
Neonatal	Passive immunity and gastrointestinal adaptation	Very high	Poor health, impaired growth
Pre-weaning	Rumen development and skeletal growth	High	Reduced nutrient utilization, slower development
Growing	Lean tissue accretion and metabolic maturation	High	Reduced frame size and muscle growth
Peripubertal	Endocrine activation and testicular development	Very high	Delayed puberty, reduced sperm production
Post-pubertal	Semen maturation	Moderate	Reduced breeding performance

## Data Availability

No new data were created or analyzed in this study. Data sharing is not applicable to this article.

## Abbreviations

The following abbreviations are used in this manuscript:

AI	Artificial intelligence
BCS	Body condition score
BHBA	β-Hydroxybutyrate
BUN	Blood urea nitrogen
FSH	Follicle-stimulating hormone
GH	Growth hormone
HPG axis	Hypothalamic–pituitary–gonadal axis
GH–IGF-1 axis	Growth hormone–insulin-like growth factor-1 axis
IGF-1	Insulin-like growth factor-1
LH	Luteinizing hormone
NEFA	Non-esterified fatty acids
VFA	Volatile fatty acid
